# Successful desensitization to etoposide in a patient after cardiac arrest

**DOI:** 10.1177/10781552241280723

**Published:** 2024-09-02

**Authors:** S. Pérez-Codesido, V. Azzi Virgini, S. Fertani, P. Jandus

**Affiliations:** 1 Division of Immunology and Allergology, University Hospitals and Medical Faculty of Geneva, Geneva, Switzerland; 2 Cabinet Médical Prilly Centre (CMPC), Route de Cossonay Prilly, Prilly, Switzerland; 3 Service d’oncologie, Hôpital de La Tour, Meyrin, Switzerland

**Keywords:** Etoposide phosphate, anaphylaxis, desensitization, systemic allergic reaction

## Abstract

**Introduction:**

Etoposide phosphate is a chemotherapeutic agent used to treat various malignant neoplasms. Hypersensitivity reactions may occur with its use, and in rare cases, an anaphylactic reaction can manifest. Available options for patients experiencing hypersensitivity reactions include premedication, changing treatment, or undergoing desensitization. Various pediatric desensitization protocols have been described, ranging from six to fifteen steps, while published adult cases are rare.

**Case Report:**

We report the case of a 61-year-old woman with small-cell lung cancer and brain metastases. In November 2019, she underwent the second cycle of cisplatin and etoposide phosphate treatment. While receiving etoposide phosphate, she experienced dyspnea and suffered a cardiorespiratory arrest, leading to cardiopulmonary resuscitation and subsequent admission to the Intensive Care Unit. Her acute tryptase levels were notably elevated at 18 µg/L (compared to a baseline tryptase level of 6,6 µg/L) during the reaction.

**Case Management:**

We implemented a 16-step desensitization protocol (without premedication) under close monitoring in an intermediate care unit. The protocol was successfully executed over three cycles until tumor progression mandated a modification in systemic treatment.

**Discussion:**

To our knowledge, this is the first documented case of successful desensitization to etoposide phosphate in a patient who experienced cardiac arrest during a hypersensitivity reaction. Although protocols of varying lengths have been published, we emphasize the importance of individualizing each protocol to fit the severity of the reaction and the resources and experience of each unit.

## Introduction

Hypersensitivity reactions (HSRs) to chemotherapeutic agents are adverse immune responses that can occur during or after the administration of chemotherapy drugs. These reactions vary in severity and can be classified into different types based on their underlying mechanisms: immediate HSR (type I), cytotoxic HSR (type II), immune complex-mediated HSR (type III), or delayed-type HSR (type IV). Antineoplastic chemotherapeutic agents can cause immediate HSRs through IgE-mediated and non-IgE- mechanisms, leading to mast cell or basophil degranulation. This can result in symptoms ranging from mild cutaneous reactions, such as urticaria, to life-threatening anaphylactic reactions.^1^ The diagnosis of anaphylaxis relies on a suggestive clinical history after exposure to a potential triggering factor. Serum tryptase concentrations increase upon degranulation of mast cells, and therefore, serum tryptase levels are measured to diagnose anaphylaxis. There is no standardized method for assessing total serum mast cell tryptase (MCT) in anaphylaxis. The Working Conference in 2010 proposed a consensus equation (peak MCT should be >1.2x baseline tryptase + 2 µg/L) to diagnose acute mast cell activation syndrome.^2^ While initial reactions such as hypotension and syncope have been documented in patients receiving drugs like carboplatin, paclitaxel, doxorubicin, rituximab, and cisplatin followed by successful desensitization to the offending drug,^3^ cases of successful desensitization to chemotherapeutic agents after cardiac arrest are rare in the literature.^4^ Here, we present the first documented case of successful desensitization to etoposide phosphate after cardiac arrest.

## Case report

We describe the case of a 61-year-old woman diagnosed with small-cell lung cancer with brain metastases in October 2019. In November 2019, she received the second cycle of cisplatin 25 mg/m^2^ (dose protocol 45 mg) and etoposide phosphate 100 mg/m^2^ (dose protocol 190 mg). She first received cisplatin 45 mg and then etoposide phosphate, only 100 mg, due to hematological toxicity. During the etoposide phosphate administration, she presented dyspnea and cardiorespiratory arrest secondary to a severe bronchospasm, necessitating cardiopulmonary resuscitation and admission in the Intensive Care Unit. Peak tryptase measured during the reaction was elevated at 18 µg/L. A second sample taken 6 h later measured 11.3 µg/L, and a third tryptase level collected 24 h after the reaction abated measured 6.6 µg/L, providing a baseline tryptase value. According to the proposed consensus equation,^2^ the peak tryptase was significantly elevated, indicating acute mast cell activation syndrome. Since anaphylactic shock typically occurs quickly after exposure to the offending drug, and the reaction occurred during the administration of etoposide phosphate, cisplatin was not considered the culprit drug.

As cisplatin was considered less probable as the culprit, it was proposed to administer another platinum agent. In January 2020, chemotherapy was switched for carboplatin and irinotecan, which was complicated with severe diarrhea.

## Case management

Due to lack of effective therapeutic alternatives to etoposide phosphate, the patient was referred to our Allergy Unit. Skin testing with etoposide phosphate was not performed because of the previous severe reaction, and an *in vitro* test was unavailable.

We proposed a 16-step desensitization protocol (without premedication) under close monitoring in an intermediate care unit (IMCU), with a total daily dose of etoposide phosphate of 100 mg (50 mg/m^2^) per day for 3 successive days. Steps 1–12 were administered in 15-min intervals. Steps 15 and 16 were part of solution 4 but were adjusted to 60-min intervals to administer doses of 25 mg and 50 mg, respectively (Table 1).

**Table 1.** Intravenous desensitization protocol for etoposide (total dose: 100 mg).

Step	Concentration (mg/mL)	Speed (mL/h)	Dose (mg)	Cumulative dose (mg)	Total time (min)
Solution1:	15 min				
1	0.001	6	0.0015	0.0015	15
2	0.001	12	0.003	0.005	30
3	0.001	25	0.006	0.01	45
4	0.001	50	0.012	0.02	60
Solution2:	15 min				
5	0.01	10	0.025	0.05	75
6	0.01	20	0.05	0.1	90
7	0.01	40	0.1	0.2	105
8	0.01	80	0.2	0.4	120
Solution3:	15 min				
9	0.1	15	0.4	0.8	135
10	0.1	30	0.8	1.5	150
11	0.1	60	1.5	3	165
12	0.1	120	3	6	180
Solution4:	15 min				
13	0.4	60	6	12	195
14	0.4	120	12	25	210
Solution4:	60 min				
15	0.4	63	25	50	270
16	0.4	125	50	100	330

Etoposide phosphate had to be reduced because of a grade 4 hematological toxicity. The desensitization protocol was thus changed for a total dose of 50 mg and successfully administered in two more cycles, until tumor progression that led to change of systemic treatment. Finally, the patient passed away (in September 2021) due to multifactorial progressive leukoencephalopathy, meningopyoventriculitis, and brain atrophy.

## Discussion

Etoposide, a chemotherapeutic agent derived from podophyllin, inhibits mitosis and is used in the treatment of various malignant neoplasms (such as small-cell lung cancer, leukemia, lymphomas, neuroblastomas and germ cell tumors). Its prodrug, etoposide phosphate, is water-soluble, and after administration, it has an identical pharmacokinetic profile to etoposide. Toxicity and clinical activity are also the same. However, because etoposide phosphate is water-soluble and can be made up to a concentration of 20 mg/mL, it can be administered as a 5-min bolus, in high doses in small volumes, and as a continuous infusion. Furthermore, it is not formulated with polyethylene glycol, polysorbate 80 (Tween), or ethanol, and does not cause acidosis when given at high doses.^5^ Hypersensitivity reactions to etoposide phosphate have been reported in 6% of patients, while the incidence of anaphylaxis is 0.7%.^4^

Management options for patients with hypersensitivity reactions include premedication, treatment change or desensitization. Desensitization is mainly performed for IgE-mediated reactions but also in cases where drug-specific IgE has not been demonstrated.^6^ Desensitization induces a temporary tolerant state, which can only be maintained by the continuous administration of the medication. Thus, for treatments like chemotherapy, which have an average interval of 4 weeks between cycles, the procedure must be repeated for every new course. In published protocols, the starting dose ranges from 1/1,000,000 to 1/10,000 of the full therapeutic dose.^7^ For patients with reactions involving respiratory or cardiac arrest, it is proposed a 16-step protocol instead of a usual 12-step protocol.^8,9^ In our case, a dilution of 1/66,666 with a fourth bag (dilution) was chosen. The use of premedication in desensitization protocols is controversial. While some authors^10,11^ recommend it, as it prevents mild symptoms that could increase patient's anxiety, others^12^ support avoiding premedication to ensure early anaphylactic symptoms or signs are rapidly recognized and treated. IMCUs, also known as high-dependency units, typically stand between regular wards and ICUs. In Switzerland, these units provide continuous monitoring and have an increased nurse-to-patient ratio compared to standard wards.^13^ These units can offer several modalities of noninvasive respiratory support or vasopressors, while patients requiring invasive mechanical ventilation are admitted to the ICU.

While pediatric desensitization protocols have been described, ranging from a six-step to a fifteen-step protocol,^14–17^ there are only a few published cases for adults. *Polistena* et al.^18^ reported a case of a 25-year-old man who developed an erythematous rash, chest tightness and difficulty breathing (corresponding to grade 3–4 anaphylaxis^19^) after the first dose of etoposide phosphate, who finally tolerated the drug with a 9-step desensitization protocol. *Gómez-Duque* et al.^20^ described a 63-year-old man who developed cutaneous redness, dizziness and dyspnea (corresponding to grade 3–4 anaphylaxis^19^) during the second dose of etoposide phosphate and achieved tolerance with a 10-step desensitization scheme. *Garcia Escallon* et al.^21^ reported a case of a 79-year-old woman who suffered from dyspnea, pruritus and hypotension (corresponding to grade 5 anaphylaxis^19^) while receiving the second cycle of etoposide phosphate, which she finally tolerated with a 14-step desensitization protocol. To date, no desensitization protocols have been described for chemotherapeutic agents after cardiac arrest.

The primary limitation in our case was the absence of skin tests, which could not be performed due to the severity of the initial reaction and the risk of provoking another reaction during testing, along with the unavailability of an in vitro test. Although cisplatin was administered first, the patient tolerated carboplatin in December, more than a month after the initial reaction, making a hypersensitivity reaction to platinum agents unlikely. Cross-reactivity between cisplatin and carboplatin is likely due to their structural similarities and shared platinum-based core.^22^ Severe hypersensitivity reactions, such as anaphylactic shock, typically occur rapidly after exposure to the offending drug, often within minutes. In most patients, symptoms manifest suddenly during intravenous administration. This suggests that the most likely cause of a severe reaction is the drug administered immediately prior to the onset of symptoms, in this case, etoposide phosphate.^23^

The Naranjo algorithm suggests a possible reaction to etoposide phosphate with a score of 7 (Supplemental File).^24^ However, the Naranjo algorithm has some limitations. The subjectivity in the evaluation of the algorithm questions and the need for clinical expertise can lead to different results. In addition, the algorithm cannot adequately assess the severity of allergic anaphylactic reactions, which can be life-threatening. The main limitation while applying the scale retrospectively was the impossibility to re-expose the patient to the suspected drug, which should not be considered in cases of anaphylaxis. Therefore, a more comprehensive assessment may be required. In 2010, the World Allergy Organization (WAO) proposed a uniform grading system to classify allergen immunotherapy systemic allergic reactions (SARs). One problem with the SAR grading systems is that they do not consider the reaction in its entirety. In two case reports involving adults treated with etoposide phosphate, distinguishing between grade 3 or 4 reactions was not feasible.^18,20^ A more uniform grading system could facilitate improved assessment and comparison of anaphylaxis in future interventions. Hence, we advocate for the adoption of a uniform grading system to classify and compare the severity of systemic HSRs.^19^ Desensitization protocols for etoposide phosphate, ranging from a six-step to a fifteen-step scheme, have been shown to be effective and safe in grade I-V reactions (modified WAO grading system^19^). Despite advancements in oncological therapies, there may be situations where the drug in question is indispensable or more efficacious than alternatives, necessitating desensitization. Unfortunately, informed consent could not be obtained despite exhaustive attempts to contact the family, so the article has been sufficiently anonymized not to cause harm to the patient/family.

## Conclusion

To our knowledge, this is the first reported case of a successful desensitization in a patient with a cardiac arrest for a chemotherapeutic agent. While various protocols have been published, it is crucial to tailor each one to the specific severity of the reaction and the available resources and expertise of each facility.

## Supplemental Material

sj-pdf-1-opp-10.1177_10781552241280723 - Supplemental material for Successful desensitization to etoposide in a patient after cardiac arrest Supplemental material, sj-pdf-1-opp-10.1177_10781552241280723 for Successful desensitization to etoposide in a patient after cardiac arrest by S. Pérez-Codesido, V. Azzi Virgini, S. Fertani and P. Jandus in Journal of Oncology Pharmacy Practice
