# Supplementary material for: Successful desensitization to etoposide in a patient after cardiac arrest
Source: J Oncol Pharm Pract. 2024 Sep 2;31(2):336–40. doi: 10.1177/10781552241280723 (PMC11898376; doi:10.1177/10781552241280723)
Supplement: sj-pdf-1-opp-10.1177_10781552241280723 - Supplemental material for Successful desensitization to etoposide in a patient after cardiac arrest [file sj-pdf-1-opp-10.1177_10781552241280723.pdf]

Adverse Drug Reaction Probability Scale (according ref. 11)

Question	Yes	No	Do Not Know	Score
1.Are there previous conclusive reports on this reaction?	1	0	0	1
2. Did the adverse event appear after the suspected drug was administered?	2	-1	0	2
3. Did the adverse event improve when the drug was discontinued or a specific antagonist was administered?	1	0	0	1
4.Did the adverse event reappear when the drug was readministered?	2	-1	0	0
5. Are there alternative causes that could on their own have caused the reaction?	-1	2	0	2
6. Did the reaction reappear when a placebo was given?	-1	1	0	0
7. Was the drug detected in blood or other fluids in concentrations known to be toxic?	1	0	0	0
8. Was the reaction more severe when the dose was increased or less severe when the dose was decreased?	1	0	0	0
9. Did the patient have a similar reaction to the same or similar drugs in any previous exposure?	1	0	0	0
10. Was the adverse event confirmed by any objective evidence?	1	0	0	1
	**Totale score**			**7**
